# Implementing a patient decision aid, a process evaluation of a large-scale pre- and post-implementation trial

**DOI:** 10.1007/s10549-020-05975-x

**Published:** 2020-10-24

**Authors:** D. B. Raphael Daniela, N. S. Russell, E. van Werkhoven, J. M. Immink, D. P. G. Westhoff, M. C. Stenfert Kroese, M. R. Stam, L. M. van Maurik, C. M. J. van Gestel, T. van der Weijden, L. J. Boersma

**Affiliations:** 1 Department of Radiation Oncology (Maastro), GROW School for Oncology and Developmental Biology, Maastricht University Medical Centre+, Dr. Tanslaan 12, 6229 ET Maastricht, The Netherlands; 2 Department of Family Medicine, CAPHRI School for Public Health and Primary Care, Maastricht University, Maastricht, The Netherlands; 3 Department of Radiotherapy, Netherlands Cancer Institute, Antoni Van Leeuwenhoek, Amsterdam, The Netherlands; 4 Department of Biometrics, Netherlands Cancer Institute, Antoni Van Leeuwenhoek, Amsterdam, The Netherlands; 5 Department of Radiation Oncology, Reinier de Graaf Hospital, Delft, The Netherlands; 6 Department of Radiation Oncology, Leiden University Medical Center, Leiden, The Netherlands; 7 Department of Radiation Oncology, Radboud University Medical Center, Nijmegen, The Netherlands; 8 Radiotherapy Group, Deventer, The Netherlands; 9 Radiotherapy Group, Arnhem, The Netherlands; 10 Department of Radiation Oncology, Amsterdam University Medical Centers, Amsterdam, The Netherlands; 11 Southwest Radiotherapy Institute, Roosendaal, The Netherlands

**Keywords:** Patient decision aid, Implementation, Breast irradiation, Shared decision-making

## Abstract

**Purpose:**

Patient decision aids (PtDAs) have been reported to have a positive influence on patients making a health care decision in trials. Nevertheless, post-trial implementation is poor. The aim of this study is to explore patient, clinician, and organizational success factors for implementing a PtDA designed for breast cancer patients, facing a decision on their radiation treatment.

**Methods:**

We performed a process evaluation within a multi-center pre- and post-implementation trial. The PtDA was incorporated as much as possible in the logistics of 13 participating centers. Tracking data were collected on PtDA use. Process characteristics were recorded by both clinicians and patients. A logistic regression method was applied to investigate which process characteristics were significantly related to the probability that patients logged in to the PtDA.

**Results:**

189 patients received the PtDA of whom140 (77%) used the PtDA. If patients received the link via the surgery department they were more likely to use the PtDA (OR 9.77 (1.28–74.51)), compared to patients that received the link via the radiation oncology department. If the report of the multidisciplinary team stated that radiation treatment “had to be discussed with the patient”, patients were more likely to use the PtDA (OR 2.29 (1.12–4.71)). Educational level was not related to the probability of PtDA use.

**Conclusions:**

We accomplished a high level of PtDA use. Patients were more likely to use the PtDA if they received the link via the surgery department and if “to be discussed with the patient” was written in the multidisciplinary team report.

## Introduction

Patient decision aids (PtDA) have been found to support the process of shared decision-making (SDM) [1–4]. In a review of 105 randomized clinical trials, Stacey et al. found that PtDAs improve quality of the decision-making process, lower decisional conflict and improve patient-clinician communication, without causing any harm [5, 6]. Despite these positive effects in clinical trials, PtDAs facilitating SDM have not been widely implemented in medical practice [7, 8]. Stacey et al. found a low uptake of PtDAs after trials, with only 21% of PtDAs being implemented, and another 7% being part of implementation studies [9].

While research on SDM and the effectiveness of PtDAs has grown rapidly over the last years [10], research on implementation of PtDAs in oncology care lags behind [11]. A lot of effort has been made to explore patient, clinician, and organization-related factors that determine the level of uptake of PtDAs but no hard conclusions can be drawn yet [7]. Frequently mentioned barriers include clinicians’ attitude toward the use of PtDAs and SDM, e.g., clinicians are less likely to use the PtDA when they lack confidence in the content of the PtDA, or if they believe that the PtDA does not fit their patient population [7, 12]. In addition, clinicians often feel that referring to a PtDA and using the PtDA during consultation might be too time consuming [7, 13–15]. Standardized referral to PtDAs prior to the decision-making consultation has therefore been identified as a facilitator for PtDA use [7]. A review by Elwyn et al. on the implementation of PtDAs added organizational barriers: the PtDA not fitting into the workflow of the clinic and lack of leadership might hinder implementation [7, 9]. This was also found in a review by Scholl et al. who reviewed organizational characteristics as barriers for the implementation of shared decision-making [8]. They pointed out the importance of taking characteristics of health care facilities into account to achieve practice changes. A comparative trial in prostate cancer patients also showed large differences in PtDA uptake with different approaches of PtDA dissemination [16].

We developed an online PtDA for breast cancer patients facing a decision in their radiation treatment (RT) [17]. RT after surgery lowers local recurrence rate but in certain groups of patients, this does not clearly translate into an improved survival rate [18–20]. The trade-off between RT accompanied with a lower local recurrence rate versus the possible side effects is therefore a preference sensitive decision [21].

We performed a multi-center, pre and post-implementation study with a pragmatic approach, adapting the implementation strategy to the logistics of the different centers (BRASA-study) The aim of the current paper is to report on the process evaluation of this study, by describing the level of uptake of the PtDA, and by analyzing whether we can identify patient, clinician, and organizational factors that are related to an increased level of uptake of the PtDA. The effects of the intervention on patient outcomes such as decision conflict will be described separately.

## Methods

We performed a process evaluation of the introduction of a PtDA within the BRASA-study (NCT03375801), a multi-center pre and post-implementation trial in which the intervention was the use of a PtDA. This trial has a pragmatic design to test the uptake and the effect of a PtDA [17, 21] in a setting conformed to normal clinical practice [22–24].

### Participants

All 19 radiation oncology centers in the Netherland were invited to participate in the study. One breast cancer radiation oncologist per center was invited through personal contact by e-mail.

Patients were recruited between October 2018 and July 2019. Eligibility criteria for study participation were female breast cancer patient of 18 years or older, with the ability to comprehend Dutch to understand the content of the PtDA and to give informed consent. The PtDA was developed for four sub-groups with a preference-sensitive indication of RT: DCIS group, low risk breast cancer group, thoracic wall irradiation group and boost/no boost group (Table 1). The multidisciplinary team (MDT) of the participating hospitals or the treating clinician determined whether the RT indication had to be discussed with the patient, and thus whether the patient was eligible for participating in the BRASA-study.

**Table 1:** The four sub-groups for whom the PtDA was developed

Patients with low risk ductal carcinoma in situ (DCIS) after breast conserving surgery deciding on whole/partial breast RT or no RT (DCIS group)
Patients with low risk invasive ductal carcinoma after breast conserving surgery deciding on whole/partial breast RT or no RT (low risk breast cancer group)
Patients with intermediate risk breast cancer after mastectomy deciding on thoracic wall RT or no RT (thoracic wall irradiation group)
Patients with intermediate risk breast cancer after breast conserving surgery deciding on whole breast RT with or without an extra boost dose to the tumor bed (boost / no boost group)

### Intervention

#### Patient decision aid (PtDA)

The PtDA is an online tool, only available in Dutch on www.beslissamen.nl, for decision-making on breast cancer RT. The PtDA was developed according to the international criteria for PtDA development (IPDAS criteria [3]), as described earlier [17]. In short, the PtDA starts with general information on SDM and an explanation on the PtDA use. The PtDA makes clear that there is a preference-sensitive decision to be made. Subsequently, general information is given on DCIS or breast cancer with links to the website of the national breast cancer association for more information. In addition, general information on the working mechanism and practical aspects of RT is given by written text and by a short animation film. The information of the rest of the PtDA is personalized for the four different sub-groups as mentioned in Table 1, such as information on possible side effects, recurrence rates, and possible survival benefit. We applied textual and graphical risk communication strategies, such as percentages, numeral frequencies, and population diagrams, in combination with clarification of uncertainties in text [17]. The PtDA also includes a section where patients are supported in constructing their treatment preferences and involvement in the SDM process. It is possible for patients to print an overview of their preferences, to bring to the consultation with their clinician. Patients can use the PtDA at home, at their own pace. The PtDA was reviewed by Vromans et al. and scored 83 points out of 100 [25].

#### Strategies to implement the PtDA

**Fig. 1:**
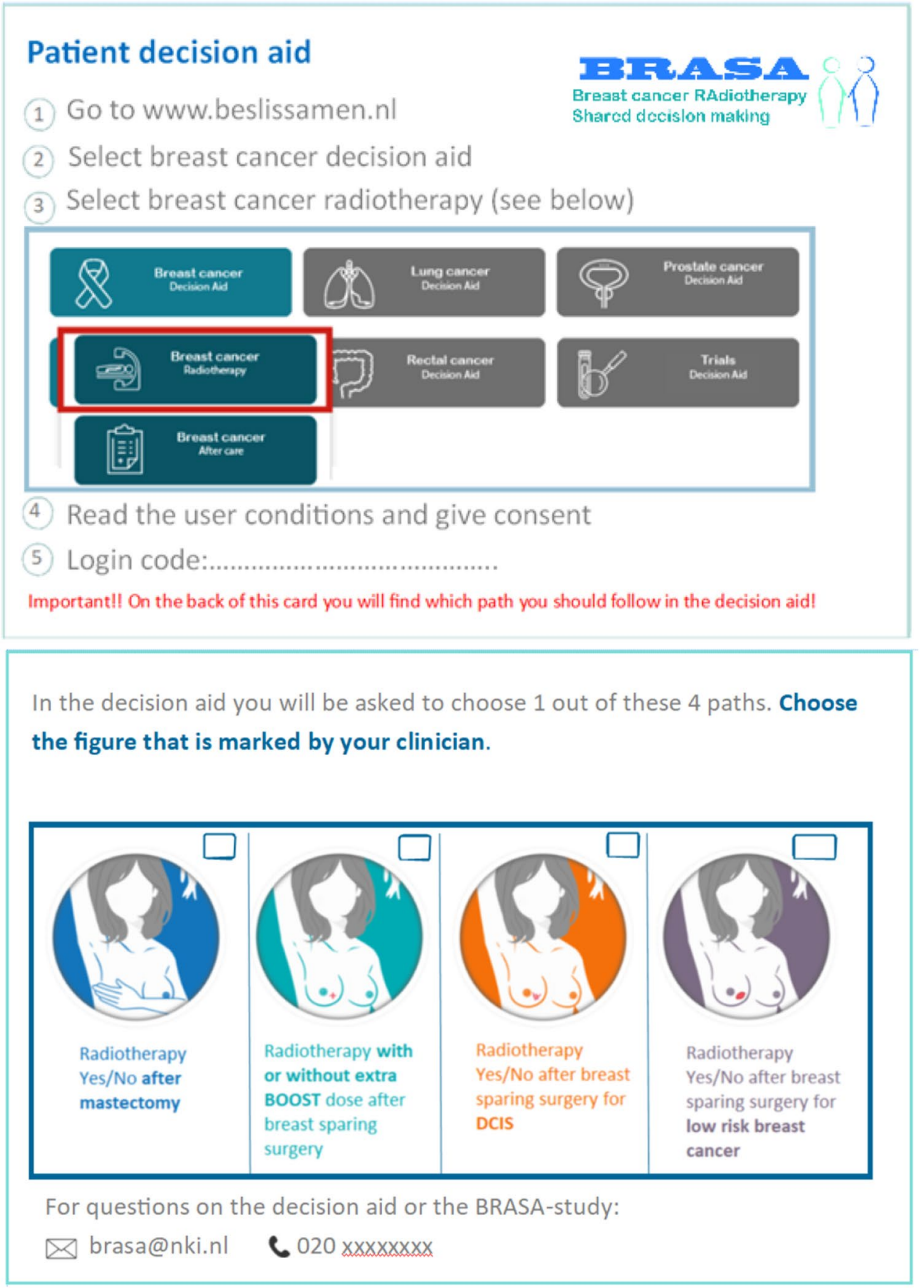
Front and backside of the recipe card

**Fig. 2:**
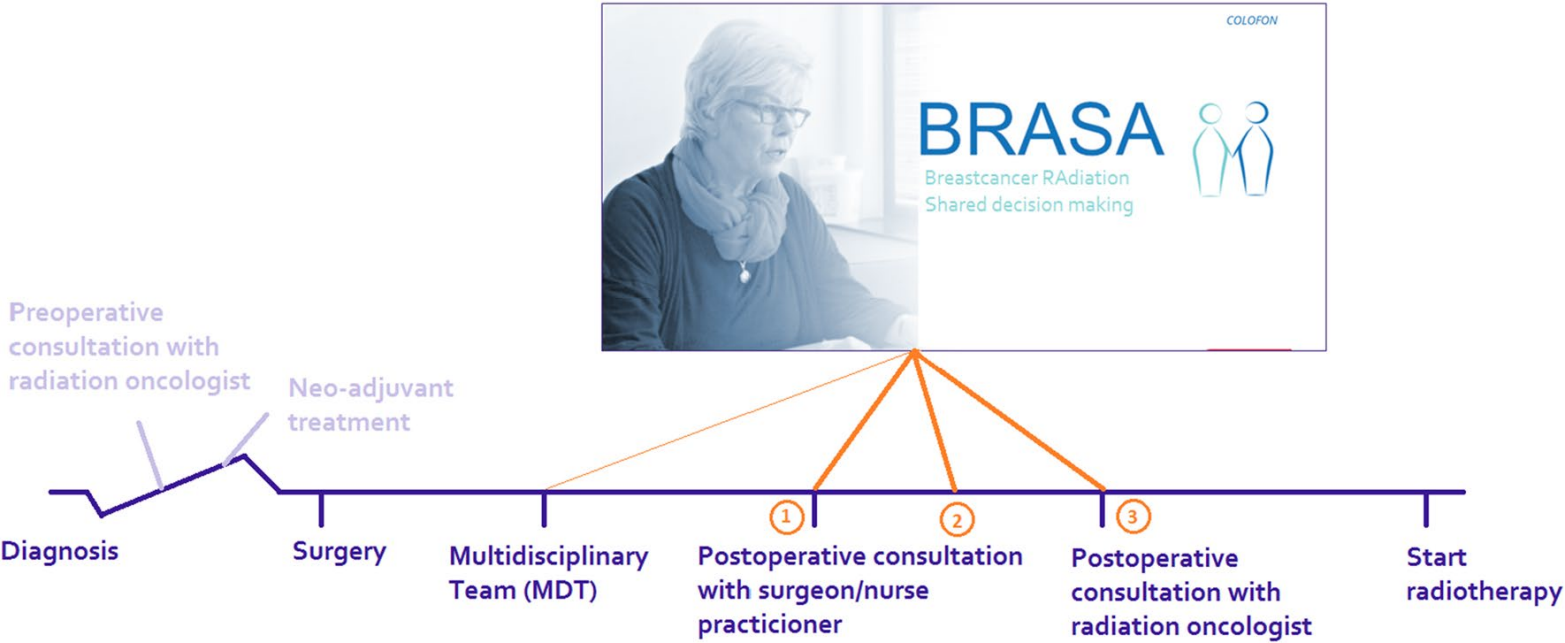
Possible moments in care path to hand over the recipe card to the patient. 1: Patients received the “recipe card” via the surgery department at the post-operative consultation; 2: patients received the “recipe card” via the radiation oncology department via regular post; and 3: patients received the “recipe card” via the radiation oncology department during the post-operative consultation

We developed so called “recipe cards” to enable the clinician to refer patients to the website with a login code. On these “recipe cards”, patients could also see which pathway they had to follow, according to their medical situation (Fig. 1). The trial logistics and PtDA use was adapted to the specific logistics and organizational preferences of each participating site. Three different moments in the care path could be distinguished to distribute the “recipe cards” in the participating centers (Fig. 2).

Via the surgery department

1. Eligible patients were identified on the post-operative multidisciplinary team meeting (MDT), and eligibility was captured in the MDT-report. Patients received the “recipe card” via the surgery department at the post-operative consultation. They referred the patient to the radiation oncologist to discuss RT and gave information about the PtDA and the trial. Patients could use the PtDA at home, prior to their consultation at the RT department.

Via the radiation oncology department

2. Eligible patients were identified on the post-operative MDT, and eligibility was captured in the MDT-report. Trial managers from the RT department sent information about the PtDA and the trial to the patient through regular post, together with the “recipe card”. Patients could use the PtDA at home, prior to their consultation at the RT department.
3. Eligible patients were informed about the study once they visited the RT department. Patients received the “recipe card” from the clinician in the RT department during the consultation. They could use the PtDA during consultation, and after the consultation at home. If necessary, a second consultation was planned.

Individual caregivers could freely choose to adapt their trial logistics for individual patients. For example, if participating centers used option 1 or 2, but patients accidentally did not receive the recipe card prior the consultation with the RT, they could follow option 3, allowing the radiation oncologist to include eligible patients anyway. In most centers, more than one of the three above7mentioned logistic options were used. Since option 2 was not foreseen when the patient questionnaire was developed, regular post was not included in the answer options on the question from who they had received the PtDA link. Consequently, we only analyzed differences between two strategies: link received via the surgical or the RT department.

### Data collection

We collected data from three sources (Table 2):

1. Tracking data: Clinicians in the participating hospitals recorded the login code of the "recipe” cards given to the patient on the case report form (CRF). When patients logged in to the website, this was automatically registered.
2. Patient questionnaires: Patients were asked to fill in a questionnaire after the consultation in which the decision was made. In this questionnaire, we asked from whom and when the patient had received the recipe card, if they had used the PtDA and if they had used the PtDA, if they perceived it as being useful. In addition, we asked some general questions e.g., on educational level, as defined by the SOI 2016 classification [26].
3. The including clinician filled in a standardized consultation registration form, part of the CRF. The CRF included questions on birth date, disease and treatment characteristics, consultation length and if there was a note in the multidisciplinary team report stating that “RT had to be discussed with the patient”.

**Table 2:** Overview of the different data to be collected

1. Log data	Log data was automatically tracked when patients logged in to the PtDA
2. Case report form to be filled in by radiation oncologist	Did the multidisciplinary board register “to discuss RT with the patient” in the multidisciplinary team report? ○ No ○ Yes ○ There is no report of the multidisciplinary team meeting
	Can you indicate the consultation length? …… min
3. Patient questionnaire	From whom did you receive the link to the decision aid? ○ Surgeon ○ Nurse/Nurse practitioner at surgery department ○ Radiation oncologist ○ Nurse/physician assistant at radiation oncology department ○ Other, being……….
	Did you consider the decision aid to be useful/did it help in the decision-making process? ○ Yes ○ Partly ○ No
	What is your highest education? ○ Primary school ○ Lower secondary education ○ Preparatory vocational education ○ Vocational education ○ Senior general secondary education ○ Pre-university education ○ Higher professional education ○ University

### Data analysis

Tracking data were used as a binary outcome. When patients logged in to the PtDA this was automatically registered. Patients were coded to have logged in to the PtDA if a login session was registered, independent of the length and the number of times patients logged in. Patients were coded as not having logged in to the PtDA if the login code was known but no login session was registered by the system. Patients were coded as missing when their login code was unknown.

Descriptive statistics were used to describe patient characteristics and information from the patient questionnaires. The question from whom they had received the link to the PtDA was recoded to three categories: (1) When patients had received the link from the surgeon or the nurse practitioner this was coded as having received the link from the surgery department; (2) Patients who received the link from the radiation oncologist or the physician assistant were coded as having received the link from the radiation department; (3) The third category was the option “other”. Educational level was recoded to low, middle and high as defined by the SOI 2016 classification [26].

Descriptive statistics were used to describe the distribution of patients between the four indications for RT and if it was indicated in the MDT-report that RT “had do be discussed with the patient”.

Univariable logistic regression was used to compare patients who had and had not logged in to the PtDA. The independent variables tested included: from whom patients received the PtDA link, educational level, whether SDM was indicated in the MDT-report and the indication for RT. We were not able to run a multivariable analysis due to the limited sample size of the categories.

## Results

**Fig. 3:**
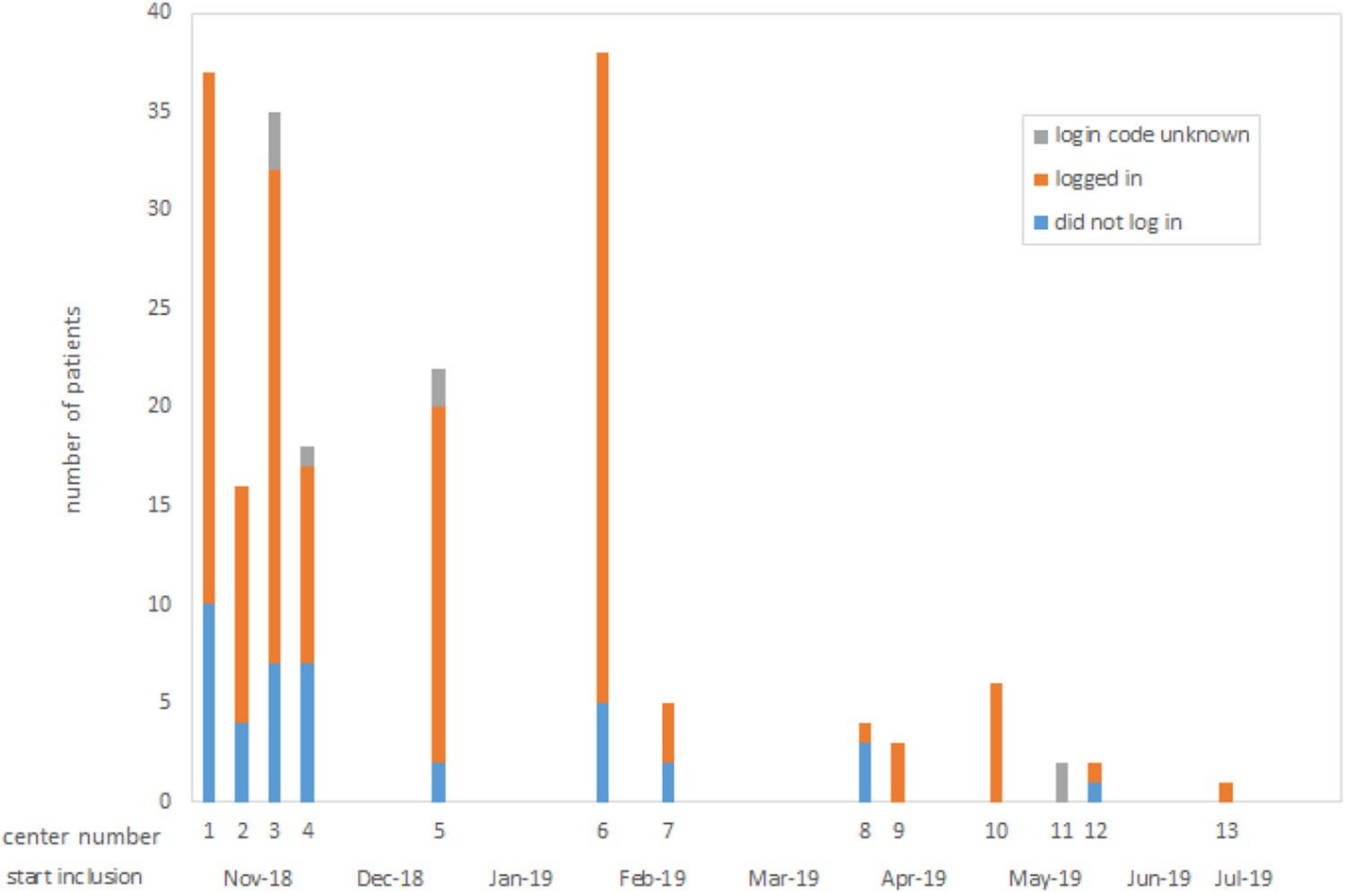
Inclusions per participating center and number of patients logging in or not. Number of inclusions per participating centers. Each bar represents one of the participating centers. Participating centers are ordered by starting date of first inclusion

14 of the 19 RT centers in the Netherlands agreed to participate in the trial of whom thirteen centers included patients. Patient inclusion started as soon as the trial was approved in each individual center, resulting in a spread in first inclusion per center (Fig. 3). There was also a spread for breast cancer patients treated per center, varying between around 1100 new patients per year in the largest center and around 200 patients in the smaller centers. 78 different clinicians included 189 patients. 185 patients filled in their questionnaire, and of 188 patients a case report form was filled in by their radiation oncologist. From eight patients the login code was unknown, such that tracking of their PtDA use was not possible. Full data were available for 181 patients.

### Patient characteristics

The mean age was 60.4 years (SD 11.3), 40% of the patients were highly educated. The low risk DCIS group was the largest group (33%), followed by the low risk invasive breast cancer group (31%) and the boost/no boost group (25%). Fewer patients deciding on thoracic wall RT were included (11%) (Table 3). Patients in the low-risk breast cancer group were the oldest group (mean 67.3 (SD 9.1)), whereas the boost/no boost group patients were the youngest (mean 54.7 (SD 11.1)).

**Table 3:** Patient characteristics of all 189 included patients

	Mean (SD)
Age in years	60.4 (11.3)
Consultation length in minutes	41.7 (13.5)

	Total N = 189
	N (%)
Received Patient decision aid via:	
Surgery department	33 (19%)
Radiation oncology department	135 (76%)
Other	10 (6%)
Missing	11
Educational level	
Low	50 (27%)
Middle	59 (32%)
High	73 (40%)
Missing	7
SDM indicated in multidisciplinary team report	
Yes	120 (66%)
No	62 (34%)
Missing	7
Indication for radiation treatment	
DCIS	62 (33%)
Low risk breast cancer	58 (31%)
Boost /no boost	47 (25%)
Thoracic wall irradiation	21 (11%)
Missing	1

	N = 140
Perceived decision aid as being useful	
Yes	55 (40%)
Partly	64 (47%)
No	17 (13%)
Missing	4

### Decision aid use and process characteristics

From the tracking data, we found that 140 (77%) patients logged in to the PtDA, whereas 41 patients did not login to the PtDA. 158 patients reported to have used the PtDA of which 136 were traced by the tracking data (Table 4). Of the patients who used the PtDA, 88% perceived the PtDA as being (partly) useful. In 114 patients (65%) eligibility had been captured in the MDT-report (Table 5). In the univariable analyses, we found that when the surgery department provided the link to the patient, patients were more likely to login to the PtDA, OR 9.77 (95% CI 1.28–74.51). In addition, when a remark in the MDT-report on the indication for SDM was found, patients were more likely to login to the PtDA, OR 2.29 (95% CI 1.12–4.71). There was no difference in login frequency between the four different sub-groups/indication for RT. Educational level did not differ between the group who did and did not login to the PtDA. There was no difference in consultation length between the group who logged in to the PtDA and those who did not (mean 41.9 min (SD 13.0) versus 40.5 min (SD 11.8).

**Table 4:** Overview of tracking data and patients answer on the questionnaire on PtDA use

	Tracking data			Total
	Did login	Did not login	Missing	
Patient questionnaire				
Did use PtDA	136	16	6	158
Did not use PtDA	2	22	2	26
Missing	2	3	0	5
Total	140	41	8	189

**Table 5:** Results of the univariable logistic regression analysis in the 181 patients for whom tracking data were available

	Did login	Did not login	OR (95%CI)	Difference
N (%)	140 (77%)	41 (23%)		
Mean age in years (SD)	59.5 (11.0)	63.4 (11.9)	0.97 (− 0.01–7.90)	3.9
Consultation length in minutes (SD)	41.9 (13.0)	40.5 (11.8)	1.00 (− 5.98–3.18)	− 1.4
Received patient decision aid via:				
Radiation oncology	98 (75%)	33 (25%)	1	
Surgery	29 (97%)	1 (3%)	9.77 (1.28–74.51)	
Other	9 (90%)	1 (10%)	3.03 (0.37–24.83)	
Missings	4	6		
Educational level				
Low	34 (72%)	13 (28%)	1	
Middle	45 (79%)	12 (21%)	1.43 (0.58–3.53)	
High	56 (80%)	14 (20%)	1.52 (0.64–3.64)	
Missings	5	2		
Indicated in multidisciplinary team report				
No	41 (67%)	20 (33%)	1	
Yes	94 (82%)	20 (18%)	2.29 (1.12–4.71)	
Missings	5	1		
Indication for radiation treatment				
Thoracic wall RT	18 (90%)	2 (10%)	1	
DCIS	47 (82%)	10 (18%)	0.52 (0.10–2.62)	
Low risk breast cancer	43 (75%)	14 (25%)	0.34 (0.07–1.66)	
Boost/no boost	32 (70%)	14 (30%)	0.25 (0.05–1.25)	
Missings	0	1		

## Discussion

The major finding in this pragmatic trial was that we reached a high level of PtDA use: 77% of the patients (140 out of 181) logged in to the PtDA [15, 27]. In addition, we found that handing out the link via the surgery department, and a remark in the MDT-report, RT is to be discussed with the patient, increased the number of patients logging in to the PtDA.

We think that the high uptake level was reached because we tackled several known barriers during trial initiation. As described in our previous paper [17], the PtDA was developed by the research team, including radiation oncologists in collaboration with clinicians from different radiotherapy centers in the Netherlands. This might have facilitated confidence in the PtDA by clinicians, which is also supported by the fact that 78 different clinicians included patients. This confidence in the intervention, or feeling of ownership, is also stated as important in the Consolidated Framework For Implementation Research (CFIR) where it is described as “commitment, involvement and accountability of leaders and managers with the implementation” [28]. Furthermore, with the pragmatic trial approach we tackled many other known barriers mentioned in the CFIR, such as logistics and leadership: by adapting the implementation of the PtDA to the logistics of the different centers, it interfered as little as possible with the routine in each center. Leadership was accomplished by having dedicated clinicians as principal investigator in each of the participating centers. In the Netherlands, there is a well-functioning national platform for breast cancer radiotherapy, which has been existing for 20 years. This mono-disciplinary platform includes at least one breast cancer radiation oncologist from every RT center, resulting in a strong cohesion. A disadvantage is that other breast cancer specialists, such as surgeons and medical oncologists are not represented. We hypothesize that this was overcome by the dedicated radiation oncologists who informed the surgeons of their referring centers about the trial.

We found in our trial that patients receiving the link to the PtDA via the surgery department were more likely to login. It is known that if the patient is given a concrete treatment advice by the surgeon, without explaining the treatment to be preference sensitive, patients might be less open for SDM since they might feel that choosing another treatment than advised by the surgeon goes against his advice [29, 30]. Therefore, when the link to the PtDA is handled via the surgery department, patients were probably more aware that they had a choice. Patients could use the PtDA prior the consultation with the radiation oncologist to be prepared for the consultation and already contemplate on what is important to them [13]. In our study though, we also had a group of patients who received the link to the PtDA via the RT department by regular post, prior the consultation, such that these patients could also use the PtDA for the preparation of the consultation. This might explain the good uptake in the RT group as well. Since we did not register whether the patients received the link per regular post, we cannot draw firm conclusions on which factor is more important: the timing of receiving the link (prior to the consultation with the radiation oncologist), or the department handing out the link (surgery department or RT department).

Capturing eligible patients by the MDT was also found to be an important facilitator. Savelberg et al.: pointed out that if the MDT-report gives an advice, favoring one of the treatment options, it might be harder for clinicians and patients to overrule this advice [30, 31]. It is not surprising that if the MDT-report did not capture the indication for the PtDA, the patient more often got the link via the radiotherapy department, since the surgeon is less involved in the indication for radiotherapy than the radiation oncologist.

The mean age of our trial population is in line with the age at diagnosis of breast cancer patients in the Netherlands [32]. Educational level did, however, differ from the general Dutch population in which, around 33% is highly educated as opposed to 40% in our trial population [33]. We found only a slightly better PtDA uptake for younger patients compared to the older patients. This also seems in line with literature which shows that older patients do want to participate in the shared decision-making process but they tend to value more the verbal communication with their clinician [34]. Vulnerable patients are known to be under-presented in health care research, and therefore probably also in this trial [35]. Although this might result in less PtDA use in patients with a lower educational level, in our study we did not find any difference in PtDA uptake by educational level. This is in line by the results of Pickles et al. who suggest that educational level does not interfere with the effect of a PtDA found in trial setting [36].

A reassuring finding was that we did not find a difference in consultation length between patients who did and did not login to the PtDA. Since perception of time and the fear for longer consultation are known to be important barriers, we hope our results might help implementation of the PtDA in clinical practice [37, 38].

A limitation to our study is that we do not know how many patients were eligible for the study but were not offered the PtDA or refused participation. There was a wide variation in amount of patients included by the different centers; this could only partly be explained by different duration of the inclusion periods, and by the big differences in the size of the different centers. Consequently, this may have resulted in overestimation of the actual PtDA use and validation in our trial.

Another limitation is that, although we found that 88% of the patients that used the PtDA perceived it as being (partly) useful, we have no further insight in the patients’ perspectives on the different logistics of receiving the link to the PtDA. Furthermore, due to technical problems, we do not have more specific tracking data on how long and how often patients logged in to the PtDA. Despite this shortcoming, the fact that we could use log data to monitor patients using the PtDA is a strength in this study, since the log data yield objective results. Objective data on uptake seem more valuable than the self-reporting of uptake as we saw in the results of the patient questionnaires that not all patients who reported to have used the PtDA had actually logged in. Patients might have not understood the question or have given a socially desirable answer when asked on PtDA use. Another strength of this trial is that it interfered as little as possible with routine medical practice. This way we were able to find factors that are related to the level of uptake of the PtDA in regular clinical practice without trial limitations. Our real life study design, with 78 different including clinicians, might facilitate post-trial implementation since Glenn et al. found that a positive personal experience and patient satisfaction motivates clinicians more to use a support tool than scientific evidence [39]. In addition, no adjustments are needed to continue using the PtDA in the participating centers after the trial. In this way, we achieved a good uptake of the PtDA when it was offered to patients. The PtDA is currently freely available and is incorporated in the website of the patient organization as proposed by Reumkens et al. [40].

## Conclusion

We accomplished a high PtDA uptake. This may be explained by the pragmatic trial design and apparent leadership in an existing network of the involved clinicians. We found that logistics facilitating the PtDA being offered via the surgery department, prior to the consultation with the radiation oncologist and a note in the MDT-report that the PtDA should be offered, resulted in high PtDA uptake.

## Data Availability

All data were anonymized by coding personal identifiers.

## Abbreviations

PtDA: Patient decision aid
MDT: Multidisciplinary team
RT: Radiation treatment
SDM: Shared decision-making
CFIR: Consolidated framework for implementation research
CRF: Case report form
